# Steroid hormones and psychological responses to soccer matches: Insights from a systematic review and meta-analysis

**DOI:** 10.1371/journal.pone.0186100

**Published:** 2017-10-12

**Authors:** Maamer Slimani, Julien S. Baker, Foued Cheour, Lee Taylor, Nicola Luigi Bragazzi

**Affiliations:** 1 Research Laboratory ‘‘Sport Performance Optimization”, National Centre of Medicine and Science in Sport (CNMSS), El Menzah, Tunisia; 2 University of the West of Scotland, Institute of Clinical Exercise and Health Science, Hamilton Campus, Hamilton, United Kingdom; 3 High Institute of Applied Biology of Médenine, Medenine, Tunisia; 4 Athlete Health and Performance Research Centre, ASPETAR, Qatar Orthopaedic and Sports Medicine Hospital, Doha, Qatar; 5 Loughborough University, School of Sport, Exercise and Health Sciences, Loughborough, United Kingdom; 6 School of Public Health, Department of Health Sciences (DISSAL), Genoa University, Genoa, Italy; 7 Department of Neuroscience, Rehabilitation, Ophthalmology, Genetics, Maternal and Child Health (DINOGMI), Section of Psychiatry, Genoa University, Genoa, Italy; Waseda University, JAPAN

## Abstract

The present systematic review and meta-analysis aimed to assess the perturbations in hormonal and psychological homeostasis in response to soccer match-play. These perturbations were explored according to match outcome (*i*.*e*., win *versus* loss), gender, type of contest (*i*.*e*., competitive *versus* non-competitive fixtures) and competitive level (*i*.*e*., novice *versus* high-level). The review was conducted according to the Population/Intervention or Exposure/Comparison/Outcome(s) (PICO) criteria and the Preferred Reporting Items for Systematic Reviews and Meta-analyses (PRISMA) guidelines. Match outcome, type of contest and competitive levels were moderator variables in the examined steroid hormones responses to a soccer match-play. Different testosterone responses were seen between match winners (increase) and losers (decrease) when compared to pre-game or baseline values (p <0.05), whilst no changes could be detected for cortisol relative to match outcome in female soccer players. Males (Δ% = 6.26; ES = 0.28) demonstrated a marginally lower increase in testosterone levels when compared to females (Δ% = 49.16; ES = 1.00), though not statistically significant. Females (Δ% = 162.7; ES = 0.98) did not demonstrate elevated cortisol match response compared to males (Δ% = 34.60; ES = 1.20). Male novice soccer match-play increased cortisol levels compared to high-level soccer match-play (Q = 18.08, p<0.001). Competitive soccer matches increased cortisol levels compared to non-competitive fixtures (i.e., collegiate tournament). Additionally, competitive levels moderate the relationship between a soccer match and testosterone levels (p <0.001), regardless of gender differences. From the presented systematic review and meta-analysis it appears (1) cortisol changes are associated with cognitive anxiety in starter female soccer players, while (2) testosterone changes are associated with changes in mood state in females and social connectedness in male soccer players. This apparent psycho-physiological relationship may proffer the opportunity for targeted intervention(s) by practitioners to favorably influence performance and/or recovery agendas. Further mechanistic and/or applied evidence is required in this regard in addition to further data sets from females.

## Introduction

Soccer is a high-intensity intermittent sport characterized by high variability within and between games [1,2]. Various contextual factors such as match score, tactics, fixture time and location, environmental conditions, League ranking, championship or cup competition, travel demands proximal to competition, amongst others, can influence match-play characteristics and post-game psycho-physiological responses [3,4]. These factors conspire to elicit psychological and physiological perturbations to homeostasis relative but not limited to, muscular, endocrine, and immune systems [5].

The adaptations of autonomic nervous system (sympathetic and parasympathetic systems) and other body systems during and response to various stressors have been extensively explored [6–8]. Adaptive changes to stressors can be classed as behavioural or physical, which can interact along with the brain and its peripheral components to simulate the hypothalamic-pituitary-adrenal axis (HPA) and the autonomic sympathetic system [5]. These systems therefore have interrelated psychological (e.g., anxiety and mood state) and physiological (e.g., cortisol and testosterone responses) components (e.g., responses are psycho-physiological in nature). Consequently, monitoring the psycho-physiological response to training and competition, including the HPA axis, hypothalamic—pituitary—gonadal (HPG) axis and hypothalamic—pituitary—thyroid (HPT) axis has been seen within soccer [6–8]. Such HPT/HPA data has been used to determine player preparedness for subsequent training and competition, and inform individualized recovery strategies [9–11].

Circulating testosterone and cortisol levels fluctuate during stressors or exercises [9]. As such, they have been used in an attempt to quantitatively determine the psycho-physiological stress/effort imposed by a soccer competition [12–14] often alongside appropriate psychometric tools and/or inventories that enable researchers/practitioners to subjectively quantify the psychological status, such as the mood states [the profile of mood states (POMS)], the perception of exertion [rating of perceived exertion (RPE: [15])] and the anxiety states (anxiety rating [16]). However, large intra- and inter-individual variability in testosterone and cortisol responses to soccer competition are seen [17], hence conflicting results within the literature [18]. This variability has been attributed to a host of physical/physiological and/or cognitive factors, including but not limited to, match-play activity profile [15,19], match-outcome [win or loss [20], type of contest (*i*.*e*., competitive *versus* non-competitive fixtures), competitive level, player coping style (psychological apparatus to deal with personal and public pressure), training status, player support network, and gender [21]. Given the variability present, it appears logical to compile and subsequently systematically review the available evidence, to determine which factors are indeed moderator/mediator variables relative to the psycho-physiological responses to a soccer match-play. If psycho-physiological relationships are apparent (e.g., increased anxiety is related to match loss), it may proffer the opportunity for targeted intervention(s) by practitioners to favorably influence performance and/or recovery agendas.

Therefore, the aim of the present systematic review and meta-analysis was to determine the effects of soccer match-play on steroid hormones (i.e., testosterone and cortisol) and psychosocial responses (mood state, competitive anxiety, psychological stress, social connectedness), identifying key moderator/mediator variables like match-outcome, gender, type of contest and competitive level. It is hypothesized that (1) match outcome, type of contest and competitive level may moderate the soccer match-hormonal responses relationship, and (2) cortisol and testosterone changes would be associated with changes in anxiety and mood state respectively.

## Methods

### Search strategy

**Fig 1:**
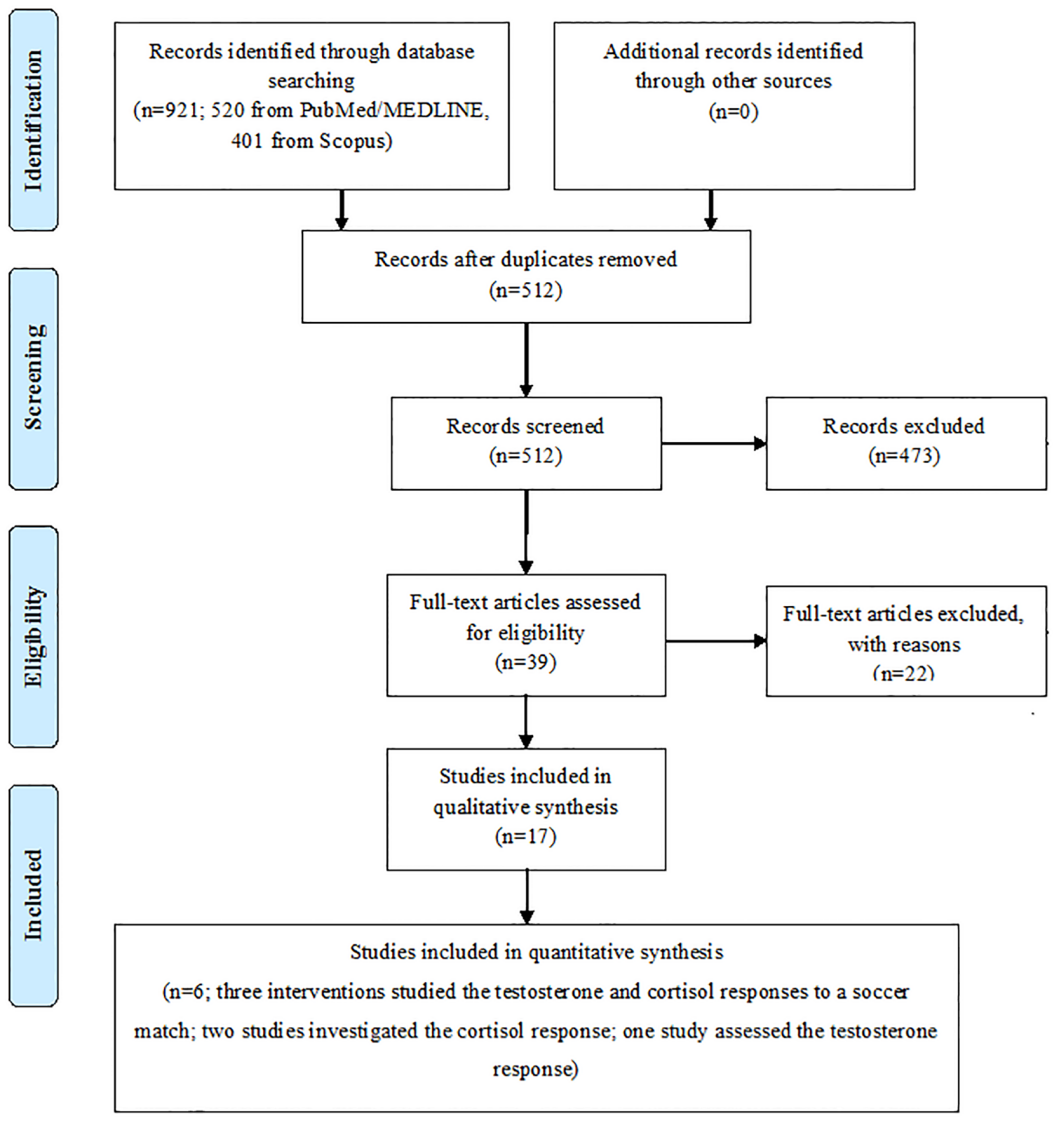
Preferred Reporting Items for Systematic Reviews and Meta-analysis (PRISMA) flow-chart.

The present systematic review and meta-analysis was conducted according to the Preferred Reporting Items for Systematic Reviews and Meta-analysis (PRISMA) guidelines [22] (Fig 1, S1 PRISMA Checklist). The systematic search was conducted using different databases, as recommended by the Cochrane Association, namely PubMed/MEDLINE, Scopus (Elsevier), SciVerse ScienceDirect (Elsevier), Institute for Scientific Information (ISI)/Web of Science (WoS), SPORTDiscus, ProQuest, Chemical Abstracts Service (CAS), the Directory of Open Access Journals (DOAJ), the Cochrane Database of Systematic Reviews (CDSR) of the Cochrane Library, the Cumulative Index to Nursing and Allied Health Literature (CINAHL), the Scientific Electronic Library Online (SciELO), and Google Scholar with dates ranging from the earliest record to April 2016. All study designs were included. The search terms included the following keywords: “soccer match”, “hormonal response”, “testosterone”, “cortisol”, and “stress”, connected using proper Boolean connectors and using Medical Subject Headings (MeSH) and wild-card options, when appropriate. Target journals have been hand-searched for capturing all potentially relevant studies.

### Inclusion and exclusion criteria

Studies were included in the review if they met all the following Population/Intervention /Comparison/Outcome(s) (PICO) criteria:

1. *Population*: studies recruiting male and female novice and/or high-level soccer players as participants.
2. *Intervention or Exposure*: investigations studying the psychological changes over the duration of a soccer match and focusing on the hormonal responses using any hormonal measurements methods and collecting blood, urine or saliva samples. Salivary and serum derived hormone values were deemed equally valid, exhibiting strong positive correlations with one another [23–26].
3. *Comparison*: steroid hormones and psychological responses to a soccer match relative to match-outcome, gender, type of contest, and competitive level.
4. *Outcome(s)*: pre- to post-steroid hormones (i.e., testosterone and cortisol) changes to a soccer match-play and the correlation between the steroid hormones response and the psychosocial status.
5. *Design*: original investigations published in scholarly, peer-reviewed journals.
6. *Time filter*: from inception to April 2016.
7. *Language filter*: English.

Studies were excluded according to the following criteria:

1. Reviews, comments, opinions and commentaries, interviews, letter to editor, editorial, posters, conference abstracts, book chapters, and books were excluded; available reviews have been anyways scanned for increasing the chance of including potentially relevant articles.
2. Focusing on methodological issues (for example, comparing endocrinological assays).
3. Comparing hormonal responses between footballers and lay people and not over soccer matches.
4. Assessing hormonal change after nutritional supplements or other kinds of intervention (interventional studies).
5. Lacking quantitative information and details.

### Screening strategy

The studies have been independently screened by two authors (MS, NLB) looking at study titles and abstracts for potential eligibility. Screening questions have been *ad hoc* developed and pilot-tested with a subset of records before implementation. Disagreement has been assessed using κ statistics and has been resolved through discussion until consensus was reached; a third reviewer (JSB) and a forth reviewer (LT) have been involved when necessary.

### Review questions

The review questions of the present review are the following:

1. What hormonal changes (i.e., testosterone and cortisol) can be induced by a soccer match-play?
2. Is there an association between steroid hormones responses and psychosocial status (e.g., mood state, anxiety, psychological stress and social connectedness) during a soccer match?
3. Which are the possible moderator and mediator variables? The research literature suggests many potential moderators/mediators of the hormonal changes-soccer match relationship such as: gender, match outcome, competitive levels and the nature of the competition itself, among others.
  - Are there differences in steroid hormones responses between genders?
  - Is there a difference between winners and losers in testosterone or cortisol responses to a soccer match?
  - Is there a difference between novice and high-level players in testosterone or cortisol response to a soccer match?
  - Is there a difference between competitiveand non-competitive fixtures matches in testosterone or cortisol response to a soccer match?
  - Has the change of cortisol or testosterone levels to a soccer match been driven by the changes in psychosocial status?

### Statistical analysis

#### Effect sizes

Effect sizes (ES) were calculated with its 95% confidence interval (CI) according to Cohen [27] and represent the difference between pre- to post- match only, means hormonal values divided by the baseline standard deviation. This method permits the determination of the magnitude of the differences or the changes between pre- to post- match for each study that provided absolute mean data and standard deviations. ES was interpreted with the following rule of thumb: ES <0.2 was defined as trivial; 0.2–0.6 was defined as small; 0.6–1.2 was defined as moderate; 1.2–2.0 was defined as large; >2.0 was defined as very large; and >4.0 was defined as extremely large. A significance level of p <0.05 was considered for all the analysis.

#### Correlation

Correlated t-tests (two-tailed), Pearson correlation coefficient, Spearman rank correlation coefficients and Pearson Product-Moment Correlation were used to determine the association (r and/or p values) between testosterone or cortisol levels and psychosocial variables in the included studies. Correlations were evaluated as follows: small (0.1–0.29), moderate (0.30–0.49), large (0.50–0.69), very large (0.70–0.89), nearly perfect (0.90–0.99), and perfect (1.0) [28].

#### Heterogeneity

Statistical heterogeneity in our systematic review and meta-analysis was assessed using the Q and I^2^ statistics. If the I^2^ was >50%, this was regarded as substantial heterogeneity. To identify sources of variation, further stratification was performed relative to the main characteristics of included studies, carrying out meta-regression analyses in order to quantitatively investigate the relationship between one or more covariates (moderators) at the study level and a dependent variable (that is to say, the effect size). In addition, for the sensitivity analyses, the stability of the pooled estimate with respect to each study was investigated by excluding individual studies from the analysis.

## Results

### Study selection

The search strategies yielded a preliminary pool of 921 possible papers. The full text of 39 articles were retrieved and assessed for eligibility against the inclusion criteria. After a careful review of their full texts, 22 articles were excluded with reason and the remaining 17 articles were eligible for inclusion in the current review (Fig 1). More specifically, 8 qualitative studies and 9 quantitative studies were noted. More specifically, qualitative study generates rich, detailed and valid process data that contribute to the in-depth understanding of a context. Quantitative study, on the other hand, generates reliable population-based and generalizable data that is suited to establishing cause-and-effect relationships. The main differences between quantitative and qualitative researches concern data sample, data collection, data analysis, and outcomes. From the quantitative studies, three interventions studied the testosterone and cortisol responses to a soccer match[4,28,29]; two trials investigated the cortisol response [16,17]; and one study assessed the testosterone response [19].

### Characteristics of included studies

In total, 17 articles were identified and retained in the present research. The characteristics of the study population included novice (i.e., healthy participants) and high-level (i.e., elite, sub-elite, semiprofessional, professional, national) participants (Tables 1 and 2). In addition, 16 studies included high-level players as sample participants and one studies used novice players as sample subjects. The total number of participants included in this review was 333 (177 males, 130 females and 26 participants’ gender was not specified). Sample size ranged between 7 and 42, with age ranging from 8 to 31 years. All studies were characterized by a cross-sectional design and performed a pre- and post-match hormonal analysis (Tables 1 and 2). Participant’s characteristics, such as gender, competitive level and age were extracted and tabulated for each selected study (Tables 1 and 2). Testosterone and cortisol percentage change values (Δ%) were derived from the nmol/L post-match value relative to the pre-match value.

**Table 1:** Qualitative studies assessed hormonal responses between pre- to post- soccer match (mean ± SD and/or relative effect %).

Study	Type of competition	Athletes characteristics (level; gender; age; n)	Collection method	Unit	Testosterone		Cortisol		T/C
					Real values	P values	Real values or relative changes	P values	
Aizawa et al. [39]	Simulated	High-level (i.e., elite); female; 20.0±0.4 years; 9	Blood/plasma	ng/dl for T	Pre 39.8±6		Pre 10±0.7		
				lg/ml for C	1^st^ day: 32±5.1		1^st^ day: 10.3±2.8		
					2^nd^ day: 39.6±5.4		2^nd^ day: 22.1±3.4		
					3^rd^ day: 35.7±4.3		3^rd^ day: 24.5±4		
Carli et al. [40]	Official	High-level (i.e., semi-professional); NR; 20–27 years; 26	Blood/plasma	ug/100 ml				↑ p<0.05	
Casanova et al. [45]	Official	High-level (i.e., elite); female; 22.85±4.2 years; 20	Salivary	ug/dl for C	Baseline		Baseline		
				pg/ml for T	8:00 h 172.4±63		8:00 h 0.73±0.23		
					11:00 h 108±33		11:00 h 0.39±0.09		
					18:00 h 78.25±40.8		18:00 h 0.35±0.27		
					Match 1 (lost)		Match 1 (lost)		
					11:00 h 63±29.4		11:00 h 0.48±0.15		
					18:00 h 50.3±24.9		18:00 h 0.46±0.12		
					Match 2 (won)		Match 2 (won)		
					11:00 h 78.1±29.1		11:00 h 0.44±0.12		
					18:00 h 57.2±21.1		18:00 h 0.39±0.14		
					Match 3 (won)		Match 3 (won)		
					11:00 h 67.6±34.5		11:00 h 0.44±0.15		
					18:00 h 48.8±22.1		18:00 h 0.44±0.15		
					Match 4 (lost)		Match 4 (lost)		
					8:00 h 63.5±28.7		8:00 h 0.83±0.20		
					13:00 h 45.6±19.58		13:00 h 0.61±0.22		
Edwards et al. [44]	Official	High-level (i.e., national); male and female; 18–22 years; 42	Salivary	ug/dl for C		Male (p<0.08)		↑ p<0.01)	
				pg/ml for T		Female ↑(p<0.01)		↑(p<0.01)	
Ispirlidis et al. [18]		High-level (i.e., elite); male; 21.1±1.2 years; 14	Blood/plasma	nmol/L for T and C		p>0.05	↑50.3%*		
Lupo et al. [41]	Official	High-level (i.e., semi-professional); male; 20–25 years; 18	Blood/plasma	ng/ml for T		45 min ↑(p<0.05)		↑(p<0.05)	
				ug/100 ml for C		90 min (p>0.05)		↑(p<0.05)	
Oliveira et al. [15]	Official	High-level (i.e., elite); female; 24.24±4.78 years; 33	Salivary	pg/ml for T		Winners↑ (p<0.001)		(p>0.05)	
				ng/ml for C		Losers (p>0.05)		(p>0.05)	
Souglis et al. [32]	Official	High-level (i.e., elite); male; NR; 18	Blood/plasma	μg/dL			105%*		

T/C: Testosterone/Cortisol ratio; NR: not reported; ↑: increased.

*: significantly different from pre-competition level at p < 0.05;

**Table 2:** Quantitative studies assessed hormonal responses between pre- to post- soccer match (mean ± SD and/or relative effect %).

Study	Type of competition	Athletes characteristics (level; gender; age; n)	Collection method	Unit	Testosterone	Cortisol	T/C
Coelho et al. [29]	College tournament	Novice (i.e., healthy student); male; 22.0±2.8 years; 10	Blood/plasma	ng/dl for T	Pre 581.2±38.8	14.2±1.3	40.9±6.1
				ug/dl for C	Imm Post 620.5±61.8	20.5±2.0*	30.2±6.7*
Gravina et al. [19]	Official	High-level (i.e., elite and sub-elite); female; elite = 25±5 years, sub-elite = 18.3±1.5 years; 28	Blood/plasma	ng /ml	Pre 0.57±0.2		
					Imm Post 0.77±0.3*		
Haneishi et al. [16]	Official	High-level (i.e., NCAA Division I); female; 18–24 years; 20	Salivary	nm/L		Starters	
						Pre 18.0±10.3	
						Imm Post 53.1±33.9*	
						Nonstarters	
						Pre 12.5±13.6	
						Imm Post 28.8±32.5*	
McHale et al. [42]	Official	High-level (i.e., elite); male; 8–10 years; 28	Salivary	ng/ml		10 min Pre 0.70±0.59	
						10 min Post 0.94±0.86	
Moreira et al. [17]	Official	High-level (i.e., elite); male; 23±4 years; 22	Salivary	ng/ml		Team A	
						Pre 7.6±4.4	
						Imm Post 12.12±6*	
						Team B	
						Pre 8.8±3.0	
						Imm Post 10.29±6.2*	
Peñailillo et al. [43]	Official	High-level (i.e., elite); male; 26±3.5 years; 9	Salivary	pg/ml for T	Pre 193.00±41.62	Pre 5.06±0.87	0.39±0.99
				ng/ml for C	Imm Post 134.00±55.4 ↓30..6%	Imm Post 5.30±1.19	0.25±0.89 ↓64.2%
Romagnoli et al. [38]	Simulated	High-level (i.e., elite); male; 17–20 years; 22	Blood/plasma	ng/ml for T	Pre 6.48±1.54	219.23±51.68	
				ug/L for C	30-min post 4.80±2.43*	210.13±81.62	
					24h post 5.04±1.91*	139.22±44.84*	
					48h post 4.47±1.70*	145.78±44.93*	
Silva et al. [36]	Official	High-level (i.e., elite); male; 22–31 years; 7	Blood/plasma	ng/ml for T and C	Pre 5.94±0.37	Pre 50.5±2.6	12.5±0.6%
					24h post 6.0±0.48	24 h post 63.6±3.0*	9.9±0.9*
					48h post 5.93±0.60	48h post 80.5 ± 9.1*	8.3±1.5*
					72h post 5.94±0.38	72 h post 52.3±3.2	11.5±0.8
Thorpe and Sunderland [4]	Official	High-level (i.e., semi-professional); male; 25±6 years; 7	Salivary	nmol/L for T and C	Pre 0.26±0.07	10.09±4.04	0.028±0.008
					Imm Post 0.37±0.10*	14.88±5.98	0.028±0.008

T/C: Testosterone/Cortisol ratio; NR: not reported; Imm: Immediately; ↑: increased; NCAA: National Collegiate Athletic Association.

*: significantly different from pre-competition level at p < 0.05;

### Pooled effect-sizes

**Fig 2:**
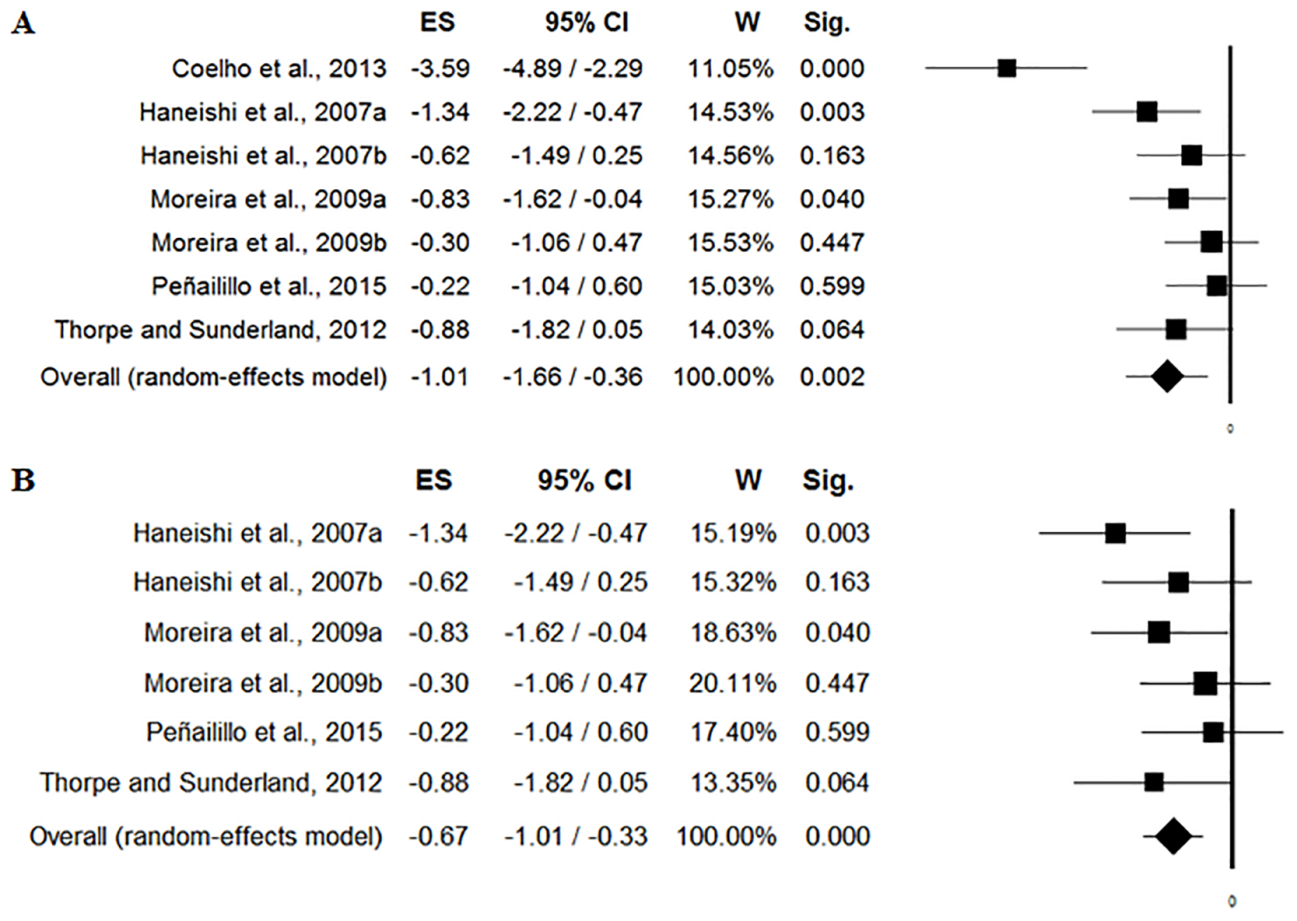
Forest plot of cortisol [nmol/L] changes induced by soccer match, including (2a) and removing (2b) Coelho et al. [29].

**Fig 3:**
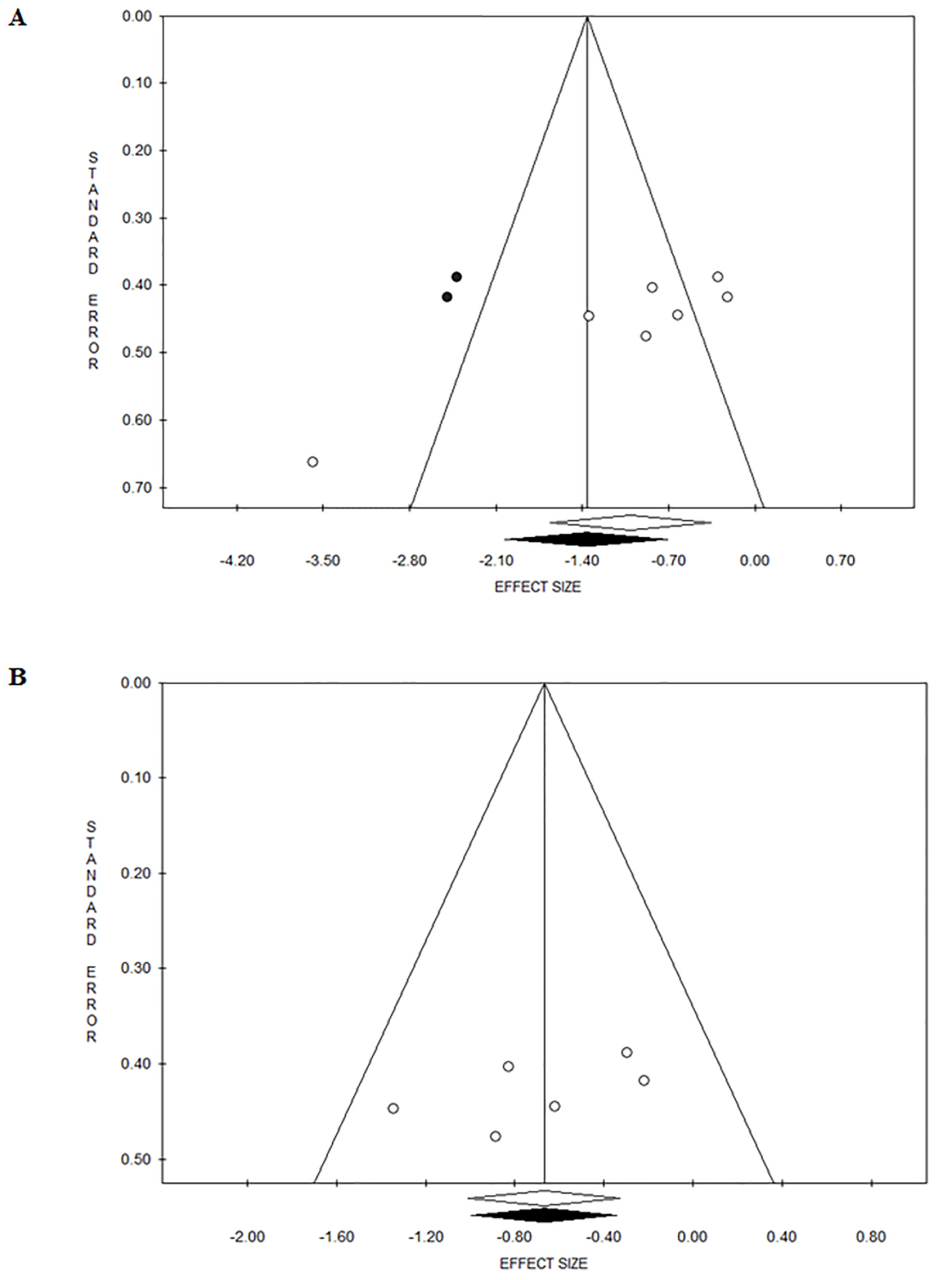
Funnel plot showing evidence of publication bias for the meta-analysis concerning cortisol changes induced by soccer match when pooling all studies, including (3a) and removing (3b) Coelho et al. [29].

The forest plot of cortisol [nmol/L] changes induced by soccer match-play is shown in Fig 2. Fixed-Effects Model Pooled ES for cortisol [nmol/L] was 1.01 ([95% CI -1.66/-0.36], p = 0.002) when including Coelho et al. [29] study’ (Fig 2a) and 0.67 ([95% CI -1.01/-0.33], p = 0.001) when removing Coelho et al. [29] study’ (Fig 2b). There was heterogeneity (Q = 22.82, I^2^ = 73.70, p = 0.001) when including Coelho et al. [29] study’ (Fig 3a), however, there was no heterogeneity (Q = 4.74, I^2^ = 0.00, p = 0.448) when removing Coelho et al. [29] study’ (Fig 3b).

**Fig 4:**
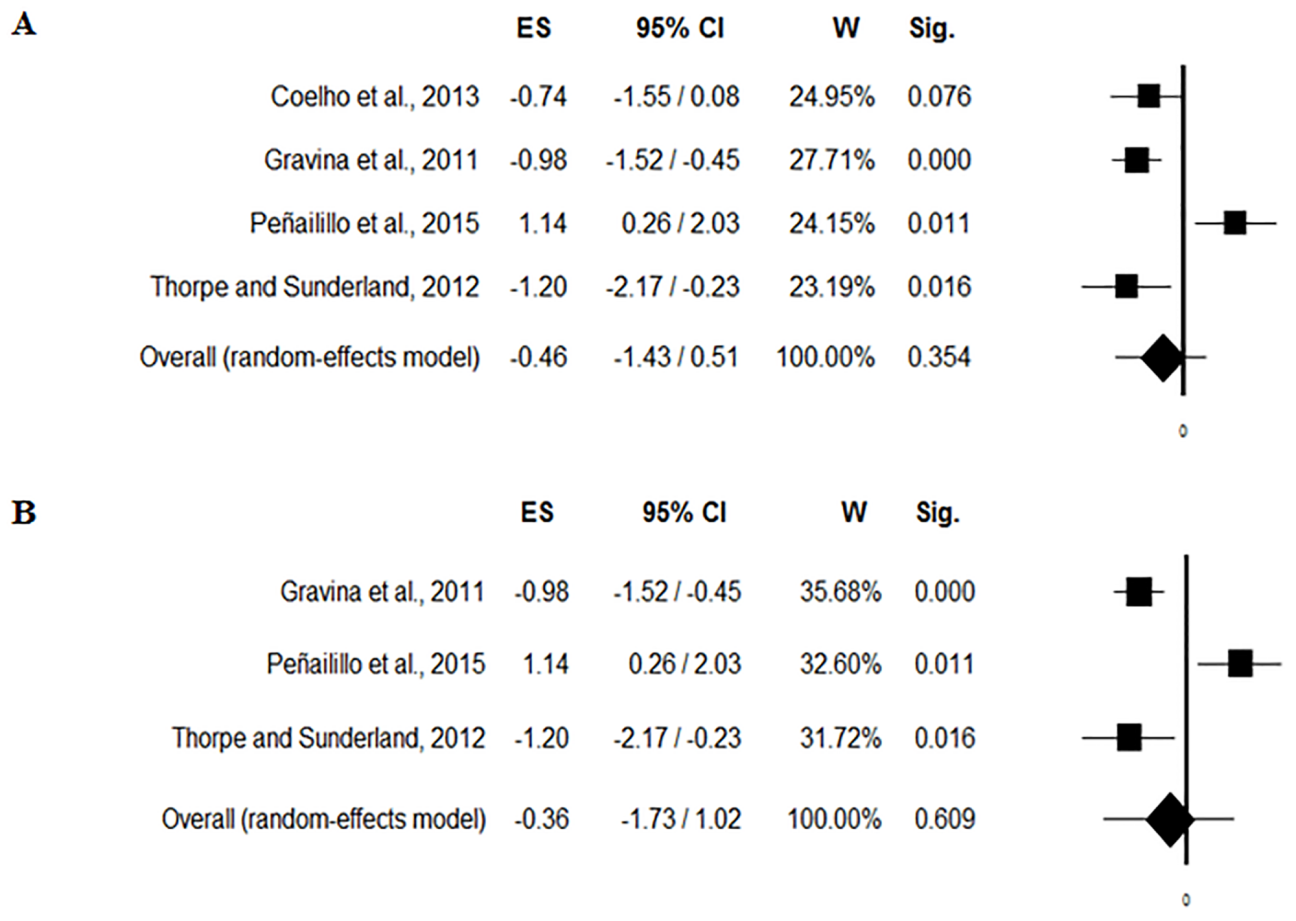
Forest plot of testosterone [nmol/L] changes induced by soccer match, including (4a) and removing (4b) Coelho et al. [29].

**Fig 5:**
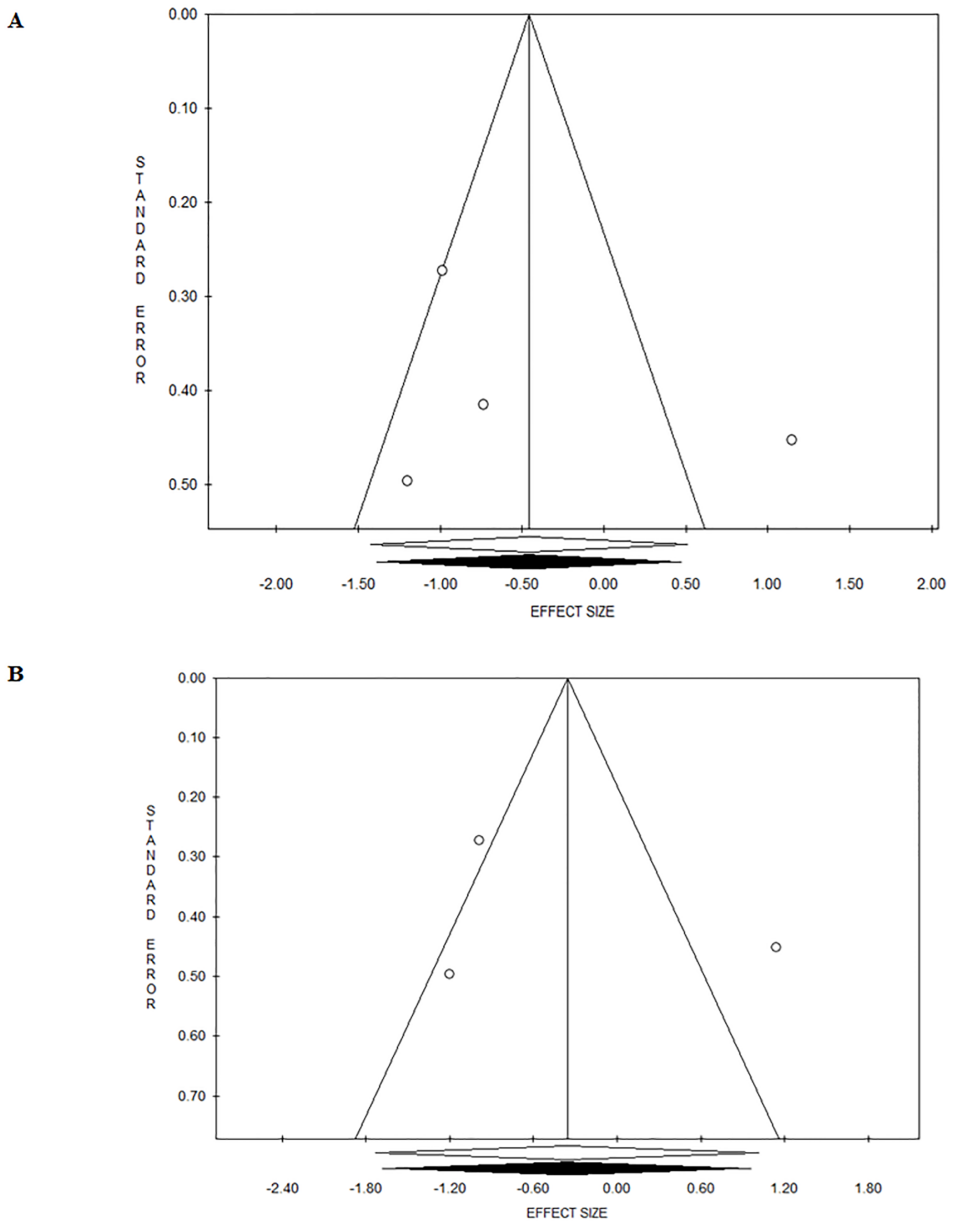
Funnel plot showing evidence of publication bias for the meta-analysis concerning testosterone changes induced by soccer match when pooling all studies, including (5a) and removing (5b) Coelho et al. [29].

The forest plot of testosterone [nmol/L] changes induced by soccer match-play is shown in Fig 4. Fixed-effects model pooled ES for testosterone [nmol/L] was 0.46 ([95% CI -1.43/0.51], p = 0.35) when including Coelho et al. [29] study’ (Fig 4a) and 0.36 ([95% CI -1.73/1.02], p = 0.60) when removing Coelho et al. [29] study’ (Fig 4b). There was heterogeneity when including Coelho et al. [29] study’ (Q = 18.42, I^2^ = 83.72, p<0.001; Fig 5a) and when removing Coelho et al. [29] study’ (Q = 18.28, I^2^ = 89.06, p<0.001; Fig 5b).

### Potential moderator and mediator variables

Male novice soccer match contest caused a large increase in cortisol levels compared to pre-match levels (Δ% = 44.36; ES = 3.73; p<0.001), the magnitude of response was significantly higher (Q = 18.08, p<0.001) than that seen in response to a high-level soccer match (Δ% = 32.16; ES = 0.57) (Table 3). Male players reported a lower percentage increase in cortisol levels (Δ% = 34.60; ES = 1.20) compared to females (Δ% = 162.7; ES = 0.98) (Table 3), even though not statistically significant. Additionally, the meta-regression analyses showed that the type of contest moderate a soccer match-cortisol response relationship (p <0.001) (Tables 4 and 5).

**Table 3:** Pre- and post- soccer match value or percentage of change (Δ%) of cortisol (nmol/L).

Study	Nature of competition	Cortisol		Δ%	ES
		Pre	Imm Post		
Novice male players					
Coelho et al. [29]	College tournament	391.92±35.88	565.8±55.2	↑44.36	3.7351
High-level male players					
Moreira et al. [17]	Official	Team A 20.97±12.14	33.45±16.56	↑59.51	0.8596
		Team B 24.28±8.28	28.40±17.11	↑16.96	0.3065
Peñailillo et al. [43]	Official	13.96±2.40	14.62±3.28	↑4.72	0.2297
Thorpe and Sunderland [4]	Official	10.09±4.04	14.88±5.98	↑47.47	0.9387
Overall				↑32.16	0.57
Overall males				↑34.60	1.20
High-level female players					
Haneishi et al. [16]	Official	Starters 18.0±10.3	53.1±33.9	↑195.0	1.401
		Nonstarters 12.5±13.6	28.8±32.5	↑130.4	0.6543
Overall females				↑162.7	0.98

ES: effect size; Imm: Immediately; ↑: increased

**Table 4:** Meta-regression analyses of studies concerning cortisol changes induced by soccer match-play, when including Coelho et al. [29].

Moderator variable	Statistical significance	Heterogeneity (ANOVA-Q)
Age	0.453	
Gender	0.897	0.02
Experience/training level	0.636	0.22
Match duration	0.853	
Type of competition/contest	0.000	18.08

**Table 5:** Meta-regression analyses of studies concerning cortisol changes induced by soccer match-play, when removing Coelho et al. [29].

Moderator variable	Statistical significance	Heterogeneity (ANOVA-Q)
Age	0.236	
Gender	0.282	1.16
Experience/training level	0.636	0.22
Match duration	0.853	

Only within competitive matches testosterone, regardless of gender and type of contest, demonstrated a small increase pre-to-post match (Δ% = 20.38; ES = 0.45), without statistical significance (Table 6). Regardless of match contest, male players reported a lower pre- to post-match percentage increase in testosterone levels (Δ% = 6.26; ES = 0.28) than females (Δ% = 49.16; ES = 1.00), without statistical significance (Table 6). A statistically significant moderator variable relationship was seen with respect to experience level of the players (p <0.001, Tables 7 and 8).

**Table 6:** Pre- and post- soccer match value or percentage of change (Δ%) of testosterone (nmol/L).

Study	Nature of competition	Testosterone		Δ%	ES
		Pre	Imm Post		
Novice male players					
Coelho et al. [29]	College tournament	20.16±1.34	21.53±2.14	↑6.79	0.77
High-level male players					
Peñailillo et al. [43]	Official	0.66±0.14	0.46±0.19	↓30.3	-1.20
Thorpe and Sunderland [4]	Official	0.26±0.07	0.37±0.10	↑42.3	1.27
Overall				↑12	0.02
Overall males				↑6.26	0.28
High-level female players					
Gravina et al. [19]	Official	1.79±0.69	2.67±1.04	↑49.16	1.00

ES = effect size; Imm: Immediately; NR: not reported; ↑: increased; ↓: decreased.

**Table 7:** Meta-regression analyses of studies concerning testosterone changes induced by soccer match-play, when including Coelho et al. [29].

Moderator variable	Statistical significance	Heterogeneity (ANOVA-Q)
Age	0.373	
Gender	0.339	0.92
Experience/training level	0.000	18.28
Type of competition/contest	0.642	0.22

**Table 8:** Meta-regression analyses of studies concerning testosterone changes induced by soccer match-play, when removing Coelho et al. [29].

Moderator variable	Statistical significance	Heterogeneity (ANOVA-Q)
Age	0.571	
Gender	0.423	0.64
Experience/training level	0.000	18.28

Finally, mood state and competitive anxiety resulted to be mediator variables of hormonal change in response to competition in soccer players. Cortisol changes were driven by changes in cognitive anxiety (very large correlation) for starter female soccer players, while testosterone changes were driven by changes in mood state (moderate correlation) for females and social connectedness (large correlation) for male soccer players (Table 9).

**Table 9:** Correlations between psychological state and cortisol and testosterone concentrations.

Study	Psychological skill	Cortisol		Testosterone	
		r	p	r	p
Edwards et al. [44]	Social connectedness (IAGSSGEQ)			Men: 0.59	<0.05
				Women: NR	>0.05
Haneishi et al. [16]	Anxiety (CSAI-2): Cognitive anxiety	Starters: 0.70	NR		
Oliveira et al.[15]	Profile of Mood States (POMS)	-0.25	0.20	0.48	<0.05
	Anxiety state	0.04	0.83	-0.36	0.06

CSAI-2: Competitive Sport Anxiety Inventory-2; IAGSSGEQ: Individual Attraction to Group-Social scale of the Group Environment Questionnaire; POMS: Profile of Mood States; RESTQ-76 Sport: Recovery-Stress Questionnaire for athletes; RPE: Ratings of Perceived Exertion. NR: not reported.

## Discussion

The aim of the current systematic review and meta-analysis was to determine the hormonal (i.e., testosterone and cortisol) responses to a soccer match according to match outcome (i.e., win, loss), gender, type of contest (*i*.*e*., competitive vs. non-competitive fixtures) and competitive level. Testosterone response was found to vary according to the game outcome, with a larger response in winners compared to losers whereas cortisol concentrations did not vary with regard to match outcome. Competitive level may have moderated the cortisol response-soccer match relationship, with greater levels of cortisol reactivity in male novice compared to high-level soccer players. Thus, competitive soccer matches increased cortisol levels to a greater magnitude compared to non-competitive fixtures (i.e., collegiate tournament). Additionally, regardless of gender differences, higher testosterone reactivity in high-level compared to novice players was shown. When psycho-physiological stress was evaluated post-match, cortisol changes appeared to be driven by changes in cognitive anxiety, while testosterone changes were driven by changes in mood state and social connectedness.

### Moderator variables

#### Match outcome and type of contest

Match outcome moderated the testosterone level in response to a soccer match (higher in winners) within the present data, in agreement with earlier meta-analyses [30]. This differential testosterone response between winners and losers proffers the opportunity for practitioners to employ a precompetitive or half-time cognitive intervention to enhance positive psychological states, such as the mood state and motivation to win, given they are related to game outcome. In contrast, cortisol increase appeared to follow general physical activity [31] with no difference between winners and losers, yet competitive compared to non-competitive matches demonstrated greater cortisol reactivity. Competitive level differences on cortisol are likely due to the higher psycho-physiological effort during competitive matches than non-competitive fixtures, specifically mental toughness, self-confidence, aerobic and anaerobic capacities [32,33]. Practitioners should therefore seek to make training sessions/matches as externally valid to competition as possible, to encourage players to develop robust coping apparatus with and familiarity to highly-competitive externally valid competition scenarios.

#### Competitive levels

It has been previously demonstrated that the competitive level is a moderator variable for the testosterone response to a soccer match, likely due to differences in players psycho-physiological competences and traits [32,33]. Increasing expertise is associated with greater physical capacities [33] and more robust psychological apparatus [32–35] in soccer players. Indeed, high-level soccer players (high-level vs. ‘other’ standards of athlete) are well acknowledged as psycho-physiologically unique [32,33]. Thus, it is worth noting that high-level players possess a high capability to cope with the game demands and stress as shown by the fast recovery pattern and lower performance impairments [36]. On the basis of the available scientific investigations, it appears that distinguishing competitive levels in regards to the hormonal responses to a soccer match-play may be useful for trainers and coaches in the development processes. More specifically, practitioners should plan specific match/training session soliciting the endocrine system in novice soccer players, in order to simultaneously decrease the psycho-physiological stress and improve the recovery pattern and performances as high-level players, and adopt the best strategy to control the stress-recovery balance.

#### Gender

It seems that gender did not moderate the hormonal response to a soccer match-play. However, this might be attributable to the small sample size and number of included studies (two studies recruiting female players; [16,19]) and so further research is warranted to determine effect of gender on hormonal responses during a soccer match. In contrast, a previous review showed that the stress response was different between males and females [37]. The differences were attributed to (a) the higher hormone concentrations for men at rest (particularly testosterone levels) (b) higher psychological stress in men immediately before the test which represents a challenge and (c) the differences in body composition (e.g., higher fat mass in women) and/or in sexual hormonal status [37].

### Hormonal changes during the recovery period after a soccer match-play

Regarding the hormonal responses during the recovery period, the competitive soccer match did not altered the testosterone level during the recovery period; however, plasma cortisol concentration significantly increased at 24 h and 48 h as compared to baseline, which returned to baseline after 72 h of recovery [36]. Furthermore, cortisol and testosterone concentrations were found to be diminished after 24 (cortisol ~ -36%, testosterone ~ -25%) and 48 h post-simulated match (cortisol ~ -32%, testosterone ~ -30%) [38]. However, in line with the findings of Silva et al. [36], the authors did not observe alterations in plasma testosterone (free testosterone) concentration throughout the 144 h of the recovery period [18]. In summary, high-level non-competitive and competitive soccer matches led to an increased catabolic hormonal environment until 48 h into the recovery period. This finding suggests that an interval of at least 72–144 h between competitions should be considered by coaches working with high-level soccer players.

### Psychological mediators

Alterations in the hormonal [39–43] and psychological variables over a soccer game are often observed, suggesting that combined psychological and hormonal changes during competition could be useful to monitor the stress in relation to soccer match performance. The assessment of psycho-physiological stress after a soccer match showed contradictory results throughout different studies. The correlation between cortisol changes and psychological states over a soccer match were investigated in five studies. Some studies showed no significant correlations between changes in cortisol over the game and changes in mood state, anxiety state in female soccer players [15], whereas only one study demonstrated a significant correlation between cortisol changes and cognitive anxiety in starter female soccer players [16].

Concerning testosterone changes after a soccer match, some studies showed significant correlations between testosterone changes and social connectedness in male soccer players [44] and mood state changes in female players [15]. In contrast, there was no significant correlation between testosterone changes, anxiety state [15] and social connectedness changes in women players [44]. For these reasons, coaches and scientists should be attentive to the role of social and environmental stress, personality, and other psychological metrics as measurable experimental variables that can influence data outcomes during a soccer match. These data also proffers the opportunity for practitioners to employ a precompetitive, or half-time, cognitive intervention that improves the participants’ mood state and consequently the participant’s chances of winning, as well as, manage the cognitive anxiety and psychosocial stress and consequently help cope both the physical and psychological demands as elite players.

### Limitations

A number of limitations affecting both the primary data and the current systematic review and meta-analysis should be properly acknowledged. First, there was a considerable amount of small numbers of included studies, particularly in female and novice players. While this review could identify important moderators of soccer match-hormonal changes, it is possible that other factors that were not assessed could also explain the observed heterogeneity. For example, training programs and time of the day when competition was played can affect the hormonal stress affecting the chronobiological system. Casanova et al. [45] observed a decrease in testosterone and cortisol levels (pre-to-post-matches), which might be explained by the circadian effect, the time of collecting samples, rather than the effect of the match *per se*. Furthermore, because of the cross-sectional design of included studies, the observed correlation between hormonal and psychological changes should not implicit as a causal relationship.

## Conclusions

This systematic review and meta-analysis provides readers with the first rigorous analytical synthesis of data concerning psychological and hormonal changes induced by soccer matches-play. In fact, the present review showed significant difference in the testosterone response to soccer games between winners and losers, with positive and negative changes in winners and losers, respectively. Furthermore, cortisol concentrations did not vary with regard to the contest outcome. Thus, it has been shown that testosterone reactivity was higher in high-level compared to novice players. Male novice soccer match contests increased cortisol levels to a greater magnitude compared to high-level soccer match. When psycho-physiological stress was evaluated after soccer matches, cortisol changes were found to be driven by changes in cognitive anxiety, while testosterone changes were driven by changes in mood state and social connectedness.

The current review highlights that match outcome and competitive levels should be considered as the key moderator variables of the soccer match-hormonal changes relationship. A psycho-physiological assessment of soccer players could give sports coaches and managers the opportunity to (1) understand the processes involved in the stress response, (2) identify how an athlete copes with stress induced by a competition, (3) reduce and mitigate ‘stress’ response of players pre, post or between games, (4) modulating the training/play load according to the specific hormonal response and (5) to design and implement various *ad hoc* mental/recovery/coping strategies for performance enhancement and optimization. Particularly, positive reevaluation and active recovery should be recommended.

## Supporting information

S1 PRISMA Checklist Preferred Reporting Items for Systematic Reviews and Meta-analysis (PRISMA) checklist [22]. (DOC)
