# Supplementary material for: Steroid hormones and psychological responses to soccer matches: Insights from a systematic review and meta-analysis
Source: PLoS One. 2017 Oct 12;12(10):e0186100. doi: 10.1371/journal.pone.0186100 (PMC5638322; doi:10.1371/journal.pone.0186100)
Supplement: S1 PRISMA Checklist — (DOC) [file pone.0186100.s001.doc]

| **Section/topic** | **#** | **Checklist item** | **Reported on page #** |
| --- | --- | --- | --- |
| **TITLE** | | |  |
| Title | 1 | Steroid hormones and psychological responses to soccer matches: insights from a systematic review and meta-analysis | 1 |
| **ABSTRACT** | | |  |
| Structured summary | 2 | The present systematic review and meta-analysis aimed to assess the perturbations in hormonal and psychological homeostasis in response to soccer match-play. These perturbations were explored according to match outcome (*i.e.*, win *versus* loss), gender, type of contest (*i.e.,* competitive *versus* non-competitive fixtures) and competitive level (*i.e.*, novice *versus* high-level). The review was conducted according to the Population/Intervention or Exposure/Comparison/Outcome(s) (PICO) criteria and the Preferred Reporting Items for Systematic Reviews and Meta-analyses (PRISMA) guidelines. Match outcome, type of contest and competitive levels were moderator variables in the examined steroid hormones responses to a soccer match-play. Different testosterone responses were seen between match winners (increase) and losers (decrease) when compared to pre-game or baseline values (p <0.05), whilst no changes could be detected for cortisol relative to match outcome in female soccer players. Males (Δ%=6.26; ES=0.28) demonstrated a marginally lower increase in testosterone levels when compared to females (Δ%=49.16; ES=1.00), though not statistically significant. Females (Δ%=162.7; ES=0.98) did not demonstrate elevated cortisol match response compared to males (Δ%=34.60; ES=1.20). Male novice soccer match-play increased cortisol levels compared to high-level soccer match-play (Q=18.08, p <0.001). Competitive soccer matches increased cortisol levels compared to non-competitive fixtures (i.e., collegiate tournament). Additionally, competitive levels moderate the relationship between a soccer match and testosterone levels (p <0.001), regardless of gender differences. From the presented systematic review and meta-analysis it appears (1) cortisol changes are associated with cognitive anxiety in starter female soccer players, while (2) testosterone changes are associated with changes in mood state in females and social connectedness in male soccer players. This apparent psychophysiological relationship may proffer the opportunity for targeted intervention(s) by practitioners to favorably influence performance and/or recovery agendas. Further mechanistic and/or applied evidence is required in this regard in addition to further data sets from females. | 2 |
| **INTRODUCTION** | | |  |
| Rationale | 3 | Large intra- and inter-individual variability in testosterone and cortisol responses to soccer competition are seen [17], hence conflicting results within the literature [18]. This variability has been attributed to a host of physical/physiological and/or cognitive factors, including but not limited to, match-play activity profile [15,19], match-outcome [win or loss [20]], type of contest (*i.e.,* competitive *versus* non-competitive fixtures), competitive level, player coping style (psychological apparatus to deal with personal and public pressure), training status, player support network, and gender [21]. Given the variability present, it appears logical to compile and subsequently systematically review the available evidence, to determine which factors are indeed moderator/mediator variables relative to the psychophysiological responses to a soccer match-play. | 3-4 |
| Objectives | 4 | The aim of the present systematic review and meta-analysis was to determine the effects of soccer match-play on steroid hormones (i.e., testosterone and cortisol) and psychosocial responses (mood state, competitive anxiety, psychological stress, social connectedness), identifying key moderator/mediator variables like match-outcome, gender, type of contest and competitive level. | 4 |
| **METHODS** | | |  |
| Protocol and registration | 5 | N/A |  |
| Eligibility criteria | 6 | Studies were included in the review if they met all the following Population/Intervention /Comparison/Outcome(s) (PICO) criteria:   1. *Population:* studies recruiting male and female novice and/or high-level soccer players as participants. 2. *Intervention or Exposure:* investigations studying the psychological changes over the duration of a soccer match and focusing on the hormonal responses using any hormonal measurements methods and collecting blood, urine or saliva samples. Salivary and serum derived hormone values were deemed equally valid, exhibiting strong positive correlations with one another [23-26]. 3. *Comparison:* steroid hormones and psychological responses to a soccer match relative to match-outcome, gender, type of contest and competitive level. 4. *Outcome(s):* pre- to post-steroid hormones (i.e., testosterone and cortisol) changes to a soccer match-play and the correlation between the steroid hormones response and the psychosocial status. 5. *Design:* original investigations published in scholarly, peer-reviewed journals. 6. *Time filter:* from inception to April 2016. 7. *Language filter:* English. | 5 |
| Information sources | 7 | The systematic search was conducted using different databases, as recommended by the Cochrane Association, namely PubMed/MEDLINE, Scopus (Elsevier), SciVerse ScienceDirect (Elsevier), Institute for Scientific Information (ISI)/Web of Science (WoS), SPORTDiscus, ProQuest, Chemical Abstracts Service (CAS), the Directory of Open Access Journals (DOAJ), the Cochrane Database of Systematic Reviews (CDSR) of the Cochrane Library, the Cumulative Index to Nursing and Allied Health Literature (CINAHL), the Scientific Electronic Library Online (SciELO), and Google Scholar with dates ranging from the earliest record to April 2016. | 4 |
| Search | 8 | The search terms included the following keywords: “soccer match”, “hormonal response”, “testosterone”, “cortisol”, and “stress”, connected using proper Boolean connectors and using Medical Subject Headings (MeSH) and wild-card options, when appropriate. Target journals have been hand-searched for capturing all potentially relevant studies. | 4-5 |
| Study selection | 9 | The present systematic review and meta-analysis was conducted according to the Preferred Reporting Items for Systematic Reviews and Meta-analysis (PRISMA) guidelines [22] (Fig 1). | 4 |
| Data collection process | 10 | The studies have been independently screened by two authors (MS, NLB) looking at study titles and abstracts for potential eligibility. Screening questions have been *ad hoc* developed and pilot-tested with a subset of records before implementation. Disagreement has been assessed using κ statistics and has been resolved through discussion until consensus was reached; a third reviewer (JSB) and a forth reviewer (LT) have been involved when necessary. | 6 |
| Data items | 11 | Pre- to post-steroid hormones (i.e., testosterone and cortisol) changes to a soccer match-play and the correlation between the steroid hormones response and the psychosocial status. | 5 |
| Risk of bias in individual studies | 12 | N/A |  |
| Summary measures | 13 | Effect sizes (ES) were calculated with its 95% confidence interval (CI) according to Cohen [27] and represent the difference between pre- to post- match only, means hormonal values divided by the baseline standard deviation. This method permits the determination of the magnitude of the differences or the changes between pre- to post- match for each study that provided absolute mean data and standard deviations. ES was interpreted with the following rule of thumb: ES <0.2 was defined as trivial; 0.2–0.6 was defined as small; 0.6–1.2 was defined as moderate; 1.2–2.0 was defined as large; >2.0 was defined as very large; and >4.0 was defined as extremely large. A significance level of p <0.05 was considered for all the analysis. | 7 |
| Synthesis of results | 14 | Statistical heterogeneity in our systematic review and meta-analysis was assessed using the Q and I^2^ statistics. If the I^2^ was >50%, this was regarded as substantial heterogeneity. | 7 |

Page 1 of 2

| **Section/topic** | **#** | **Checklist item** | **Reported on page #** |
| --- | --- | --- | --- |
| Risk of bias across studies | 15 | N/A |  |
| Additional analyses | 16 | To identify sources of variation, further stratification was performed relative to the main characteristics of included studies, carrying out meta-regression analyses in order to quantitatively investigate the relationship between one or more covariates (moderators) at the study level and a dependent variable (that is to say, the effect size). In addition, for the sensitivity analyses, the stability of the pooled estimate with respect to each study was investigated by excluding individual studies from the analysis. | 7-8 |
| **RESULTS** | | |  |
| Study selection | 17 | The search strategies yielded a preliminary pool of 921 possible papers. The full text of 39 articles were retrieved and assessed for eligibility against the inclusion criteria. After a careful review of their full texts, 22 articles were excluded with reason and the remaining 17 articles were eligible for inclusion in the current review (Figure 1). More specifically, 8 qualitative studies and 9 quantitative studies were noted. From the quantitative studies, three interventions studied the testosterone and cortisol responses to a soccer match [4,28,29]; two trials investigated the cortisol response [16,17]; and one study assessed the testosterone response [19]. | 8 |
| Study characteristics | 18 | In total, 17 articles were identified and retained in the present research. The characteristics of the study population included novice (i.e., healthy participants) and high-level (i.e., elite, sub-elite, semiprofessional, professional, national) participants (Tables 1a and 1b). In addition, 16 studies included high-level players as sample participants and one studies used novice players as sample subjects. The total number of participants included in this review was 333 (177 males, 130 females and 26 participants’ gender was not specified). Sample size ranged between 7 and 42, with age ranging from 8 to 31 years. All studies were characterized by a cross-sectional design and performed a pre- and post-match hormonal analysis (Tables 1a and 1b). Participant’s characteristics, such as gender, competitive level and age were extracted and tabulated for each selected study (Tables 1a and 1b). Testosterone and cortisol percentage change values (Δ%) were derived from the nmol/L post-match value relative to the pre-match value (Tables 2 and 4). | 8 |
| Risk of bias within studies | 19 | N/A |  |
| Results of individual studies | 20 | Tables 1 and 2. |  |
| Synthesis of results | 21 | Pooled effect-sizes  The forest plot of cortisol [nmol/L] changes induced by soccer match-play is shown in Fig 2. Fixed-Effects Model Pooled ES for cortisol [nmol/L] was 1.01 ([95% CI -1.66/-0.36], p=0.002) when including Coelho et al. [29] study’ (Fig 2a) and 0.67 ([95% CI -1.01/-0.33], p=0.001) when removing Coelho et al. [29] study’ (Fig 2b). There was heterogeneity (Q=22.82, I2=73.70, p=0.001) when including Coelho et al. [29] study’ (Fig 3a), however, there was no heterogeneity (Q=4.74, I2=0.00, p=0.448) when removing Coelho et al. [29] study’ Fig 3b).  The forest plot of testosterone [nmol/L] changes induced by soccer match-play is shown in Fig 4. Fixed-effects model pooled ES for testosterone [nmol/L] was 0.46 ([95% CI -1.43/0.51], p=0.35) when including Coelho et al. [29] study’ (Fig 4a) and 0.36 ([95% CI -1.73/1.02], p=0.60) when removing Coelho et al. [29] study’ (Fig 4b). There was heterogeneity when including Coelho et al. [29] study’ (Q=18.42, I2=83.72, p <0.001; Fig 5a) and when removing Coelho et al. [29] study’ (Q=18.28, I2=89.06, p <0.001; Fig 5b).  Potential moderator and mediator variables  Male novice soccer match contest caused a large increase in cortisol levels compared to pre-match levels (Δ%=44.36; ES=3.73; p <0.001), the magnitude of response was significantly higher (Q=18.08, p <0.001) than that seen in response to a high-level soccer match (Δ%=32.16; ES=0.57) (Table 3). Male players reported a lower percentage increase in cortisol levels (Δ%=34.60; ES=1.20) compared to females (Δ%=162.7; ES=0.98) (Table 3), even though not statistically significant. Additionally, the meta-regression analyses showed that the type of contest moderate a soccer match-cortisol response relationship (p <0.001) (Tables 4 and 5).  Only within competitive matches testosterone, regardless of gender and type of contest, demonstrated a small increase pre-to-post match (Δ%=20.38; ES=0.45), without statistical significance (Table 6). Regardless of match contest, male players reported a lower pre- to post-match percentage increase in testosterone levels (Δ%=6.26; ES=0.28) than females (Δ%=49.16; ES=1.00), without statistical significance (Table 6). A statistically significant moderator variable relationship was seen with respect to experience level of the players (p <0.001, Tables 7 and 8). | 9-10 |
| Risk of bias across studies | 22 | N/A |  |
| Additional analysis | 23 | Mood state and competitive anxiety resulted to be mediator variables of hormonal change in response to competition in soccer players. Cortisol changes were driven by changes in cognitive anxiety (very large correlation) for starter female soccer players, while testosterone changes were driven by changes in mood state (moderate correlation) for females and social connectedness (large correlation) for male soccer players (Table 9). | 10-11 |
| **DISCUSSION** | | |  |
| Summary of evidence | 24 | Testosterone response was found to vary according to the game outcome, with a larger response in winners compared to losers whereas cortisol concentrations did not vary with regard to match outcome. Competitive level may have moderated the cortisol response-soccer match relationship, with greater levels of cortisol reactivity in male novice compared to high-level soccer players. Thus, competitive soccer matches increased cortisol levels to a greater magnitude compared to non-competitive fixtures (i.e., collegiate tournament). Additionally, regardless of gender differences, higher testosterone reactivity in high-level compared to novice players was shown. When psychophysiological stress was evaluated post-match, cortisol changes appeared to be driven by changes in cognitive anxiety, while testosterone changes were driven by changes in mood state and social connectedness. | 11 |
| Limitations | 25 | A number of limitations affecting both the primary data and the current systematic review and meta-analysis should be properly acknowledged. First, there was a considerable amount of small numbers of included studies, particularly in female and novice players. While this review could identify important moderators of soccer match-hormonal changes, it is possible that other factors that were not assessed could also explain the observed heterogeneity. For example, training programs and time of the day when competition was played can affect the hormonal stress affecting the chronobiological system. Casanova et al. [36] observed a decrease in testosterone and cortisol levels (pre-to-post-matches), which might be explained by the circadian effect, the time of collecting samples, rather than the effect of the match *per se*. Furthermore, because of the cross-sectional design of included studies, the observed correlation between hormonal and psychological changes should not implicit as a causal relationship. | 14 |
| Conclusions | 26 | This systematic review and meta-analysis provides readers with the first rigorous analytical synthesis of data concerning psychological and hormonal changes induced by soccer matches-play. In fact, the present review showed significant difference in the testosterone response to soccer games between winners and losers, with positive and negative changes in winners and losers, respectively. Furthermore, cortisol concentrations did not vary with regard to the contest outcome. Thus, it has been shown that testosterone reactivity was higher in high-level compared to novice players. Male novice soccer match contests increased cortisol levels to a greater magnitude compared high-level soccer match. When psycho-physiological stress was evaluated after soccer matches, cortisol changes were found to be driven by changes in cognitive anxiety, while testosterone changes were driven by changes in mood state and social connectedness.  The current review highlights that match outcome and competitive levels should be considered as the key moderator variables of the soccer match-hormonal changes relationship. A psycho-physiological assessment of soccer players could give sports coaches and managers the opportunity to (1) understand the processes involved in the stress response, (2) identify how an athlete copes with stress induced by a competition, (3) reduce and mitigate ‘stress’ response of players pre, post or between games, (4) modulating the training/play load according to the specific hormonal response and (5) to design and implement various *ad hoc* mental/recovery/coping strategies for performance enhancement and optimization. Particularly, positive reevaluation and active recovery should be recommended. | 14-15 |
| **FUNDING** | | |  |
| Funding | 27 | The authors would like to declare that no sources of funding were used in the preparation of this review. |  |

*From:*  Moher D, Liberati A, Tetzlaff J, Altman DG, The PRISMA Group (2009). Preferred Reporting Items for Systematic Reviews and Meta-Analyses: The PRISMA Statement. PLoS Med 6(7): e1000097. doi:10.1371/journal.pmed1000097

For more information, visit: **www.prisma-statement.org**.

Page 2 of 2
